# Case Report: A Novel *COL1A1* Missense Mutation Associated With Dentineogenesis Imperfecta Type I

**DOI:** 10.3389/fgene.2021.699278

**Published:** 2021-06-23

**Authors:** Yuting Zeng, Yuhua Pan, Jiayao Mo, Zhiting Ling, Lifang Jiang, Fu Xiong, Wenjuan Yan

**Affiliations:** ^1^Department of Stomatology, Nanfang Hospital, Southern Medical University, Guangzhou, China; ^2^Department of Medical Genetics, School of Basic Medical Sciences, Southern Medical University, Guangzhou, China; ^3^Guangdong Provincial Key Laboratory of Single Cell Technology and Application, Guangzhou, Guangdong, China

**Keywords:** dentin, type I collagen, missense mutation, odontoblasts, case report

## Abstract

**Background:** Osteogenesis imperfecta (OI) is a clinical and genetic disorder that results in bone fragility, blue sclerae and dentineogenesis imperfecta (DGI), which is mainly caused by a mutation in the *COL1A1* or *COL1A2* genes, which encode type I procollagen.

**Case Report:** A missense mutation (c.1463G > C) in exon 22 of the *COL1A1* gene was found using whole-exome sequencing. However, the cases reported herein only exhibited a clinical DGI-I phenotype. There were no cases of bone disease or any other common abnormal symptom caused by a *COL1A1* mutation. In addition, the ultrastructural analysis of the tooth affected with non-syndromic DGI-I showed that the abnormal dentine was accompanied by the disruption of odontoblast polarization, a reduced number of odontoblasts, a reduction in hardness and elasticity, and the loss of dentinal tubules, suggesting a severe developmental disorder. We also investigated the odontoblast differentiation ability using dental pulp stem cells (DPSCs) that were isolated from a patient with DGI-I and cultured. Stem cells isolated from patients with DGI-I are important to elucidate their pathogenesis and underlying mechanisms to develop regenerative therapies.

**Conclusion:** This study can provide new insights into the phenotype-genotype association in collagen-associated diseases and improve the clinical diagnosis of OI/DGI-I.

## Introduction

Dentineogenesis imperfecta (DGI) is a rare autosomal dominant disease that is traditionally classified as DGI-I, DGI-II, or DGI-III, which represent hereditary developmental conditions that affect the composition and structure of dentine (Turkkahraman et al., 2020). While types II and III involve only the teeth, type I is the dental manifestation of osteogenesis imperfecta (OI), which is a connective tissue disorder characterized by osteopenia, which may be associated with blue sclerae, DGI, and hearing loss. Generally, OI is divided into type I, type II, type III, and type IV, ranging from very mild cases with nearly no fractures, to variable skeletal deformities, intrauterine fractures, and even perinatal death (Kantaputra et al., 2018; Ibrahim et al., 2019; Zhai et al., 2019).

OI can affect the life quality of patients with the disease because the main causative gene, *type I collagen* (*COL1A1*), encodes a major structural protein of dentine, bone, and other fibrous tissues (Wang et al., 2019; Li et al., 2020). Therefore, a mutation in type I collagen gene *COL1A1* gene might alter the collagen fibrils, which may affect the formation and stability of bone and dentine minerals and, finally, result in a variety of abnormal phenotypes (Marom et al., 2020). Although a lot of *type I collagen* gene mutations have been reported, DGI without OI has never been linked with *COL1A1* mutations (Zhang et al., 2016; Liu et al., 2017), and little is known on the phenotype changes of dentine structure and ultrastructure in patients with DGI-I (Orsini et al., 2014; Eimar et al., 2016; Lignon et al., 2017).

Human dental pulp stem cells (DPSCs) can differentiate into odontoblasts, and their normal differentiation is important for dentine formation; therefore, they, constitute a valuable model to investigate odontoblastic differentiation (Gronthos et al., 2000). The main pathological feature of DGI-I is the dentine mineralization abnormality. Mineralization depends on bone homeostasis and on the normal differentiation of human DPSCs (Gronthos et al., 2000; Xin et al., 2018). Moreover, DPSCs are a highly considered option for odontogenesis and pulp tissue repair (Alongi et al., 2010). Interestingly, the potential functional roles that DPSCs may have during dentine development in patients with DGI-I have not yet been studied. Therefore, human DPSCs can be a valuable model to investigate odontoblastic differentiation, which is affected by a *COL1A1* mutation.

Here, we describe a patient who had a heterozygous *COL1A1* novel mutation [c.1463G>C (p.G488A)]. Notably, she did not have any bone problems or other phenotypes associated with OI, only presenting a clinically evident DGI phenotype such as opalescent teeth, obliterated pulp chambers and marked cervical constriction of the bulbous crowns. We elucidated the ultrastructural morphological alterations of defective dentine in patients with DGI-I. Meanwhile, we investigated the characteristics of DPSCs derived from a patient with DGI-I *in vitro* to possibly develop regenerative therapies in the future. In this study, we report that a *COL1A1* mutation causes non-syndromic human DGI-I and provide a theoretical basis for the development of DPSC-based cell therapies.

## Case Description

**Figure 1:**
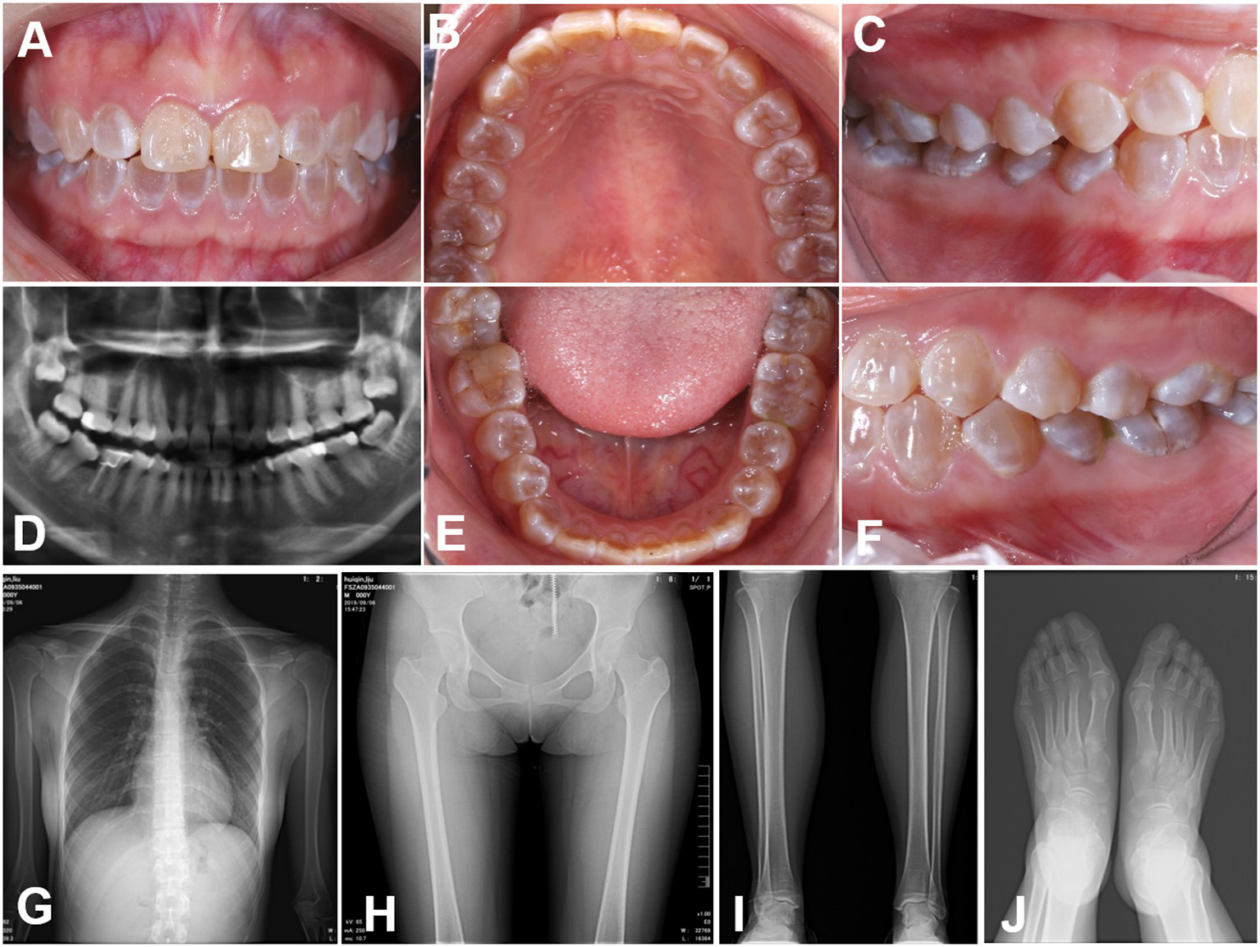
Clinical images **(A–E)** Intraoral views of the proband. The teeth of the proband were typically amber and translucent and showed significant attrition, especially the molar teeth. **(F–J)** Panoramic radiographs and radiovisiography images. The pulp chambers and root canals of the affected teeth were smaller than those in normal teeth or completely obliterated. Radiographs of the bones and knees revealed no significant osteopenia, bony destruction, periosteal reactions, or evidence of any acute fractures, dislocations or injuries.

The teeth in the proband were typically amber and translucent and showed significant attrition, especially in the molar teeth (Figures 1A–F). Radiographic examination of the teeth showed bulbous crowns with prominent cervical constriction. The pulp chambers and root canals of the affected teeth were smaller than the normal control or even completely obliterated (Figure 1D). Radiographs of limb bones and knees revealed no significant osteopenia or any acute fractures, dislocations, or injuries (Figures 1G–J). Moreover, the bone mineral density, serum calcium and alkaline phosphatase levels, sclera observation, and echocardiography revealed no remarkable findings. All the clinical characteristics and radiographic results supported a clinical diagnosis of DGI-I.

**Figure 2:**
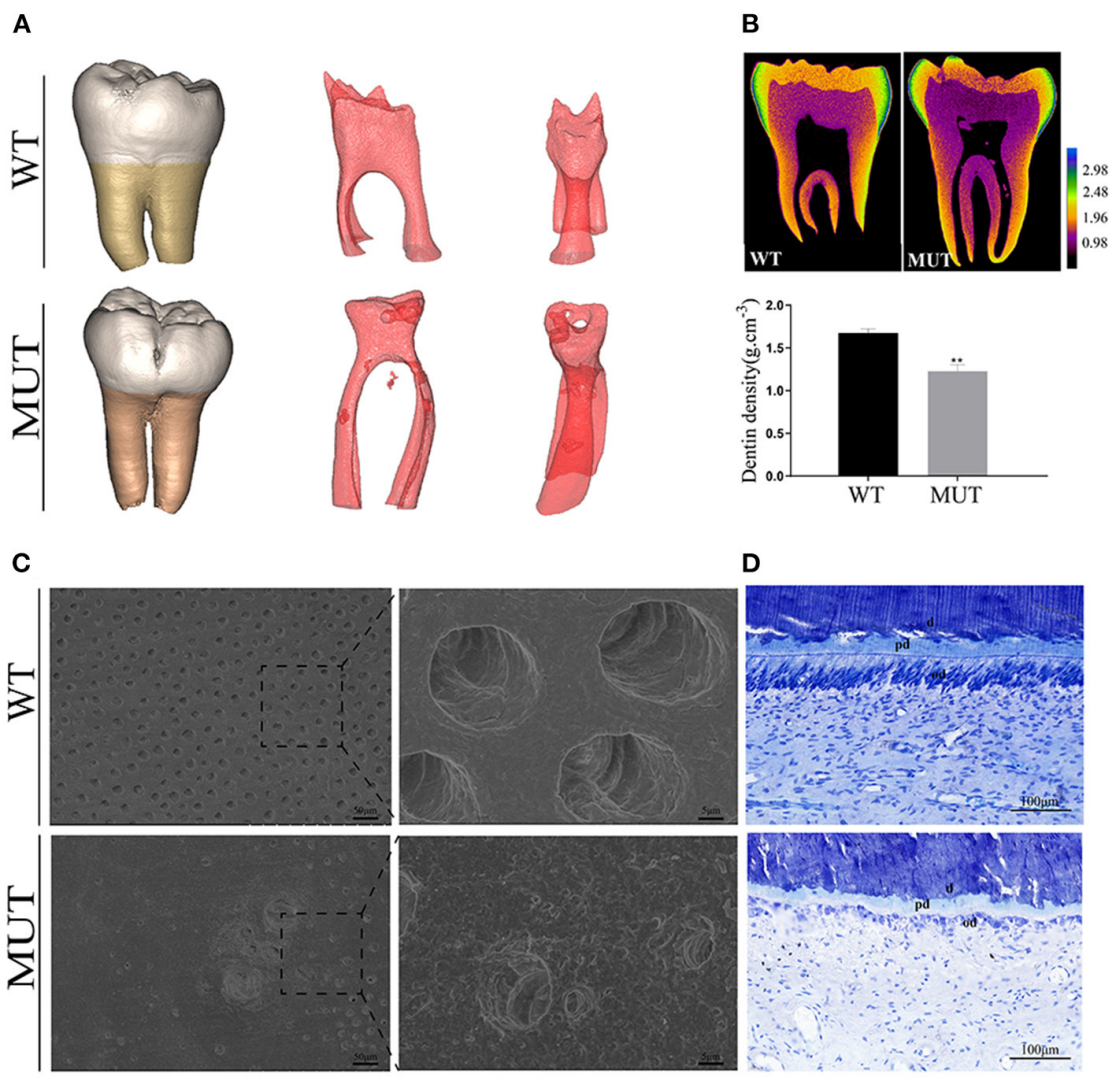
Teeth ultrastructural analyses. **(A)** 3D reconstruction of the teeth CT data. 3D reconstruction of the pulp chambers. **(B)** Typical CT sections of the teeth are presented using false colors calibrated based on the mineral density to generate mineral density maps. **(C)** SEM of representative exfoliated teeth. The SEM images of the control dentine showed regularly organized dentine tubes and an evenly calcified matrix, while the teeth of the patients with DGI-I presented very few dentine tubules and enlarged malformed dentine tubes. **(D)** Toluidine blue staining of teeth. The number and morphology of odontoblasts adjacent to the mineralized dentine layer were visibly different. d, dentine; od, odontoblast; pd, predentine.

Micro-CT analysis showed a bulbous shape and color change in the proband teeth (Figure 2A), whereas the 3D image of the pulp presented an irregularly obliterated pulp chamber and scattered pulp stones. Mineral density analysis showed that the DGI-I teeth had similar enamel mineral density, but lower dentine mineral density compared with the control teeth (Figure 2B). The SEM images of the control dentine showed the regularly organized dentine tubes and an evenly calcified matrix, while the DGI-I teeth presented very few dentine tubules and enlarged malformed dentine tubules (Figure 2C). Toluidine blue staining showed that the number of odontoblasts adjacent to the mineralized dentine layer was significantly reduced and that the odontoblast morphology was changed. The roof odontoblasts of the control teeth were columnar in shape, with the nucleus located at the basal end of each odontoblast. However, in the teeth of the patient, the odontoblasts became flattened as a result of lost polarity, and the odontoblast layer was disorganized (Figure 2E).

The nanoindentation test showed the nanoindentation load-displacement curves of the enamel and dentine, which indicated that the dentine hardness values and elastic modulus of the DGI-I teeth were significantly reduced compared to those of the control (Supplementary Figure 1). However, there was no difference in the enamel value. Energy-dispersive spectroscopy data analysis showed that the P concentration values were lower in the DGI-I teeth than in the control teeth, whereas there were no differences in the concentration values of Na, Mg, and Ca.

**Figure 3:**
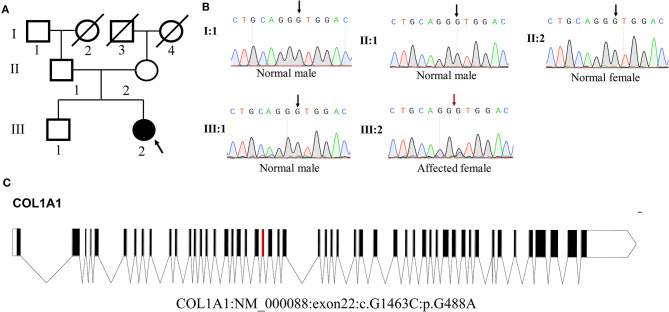
Analysis of *COL1A1* mutation. **(A)** Pedigree of the family. **(B)** Sequence chromatogram of the affected individual (heterozygous) and control (wild type). **(C)** Genomic structure of *COL1A1*. The rectangles represent exons.

**Figure 4:**
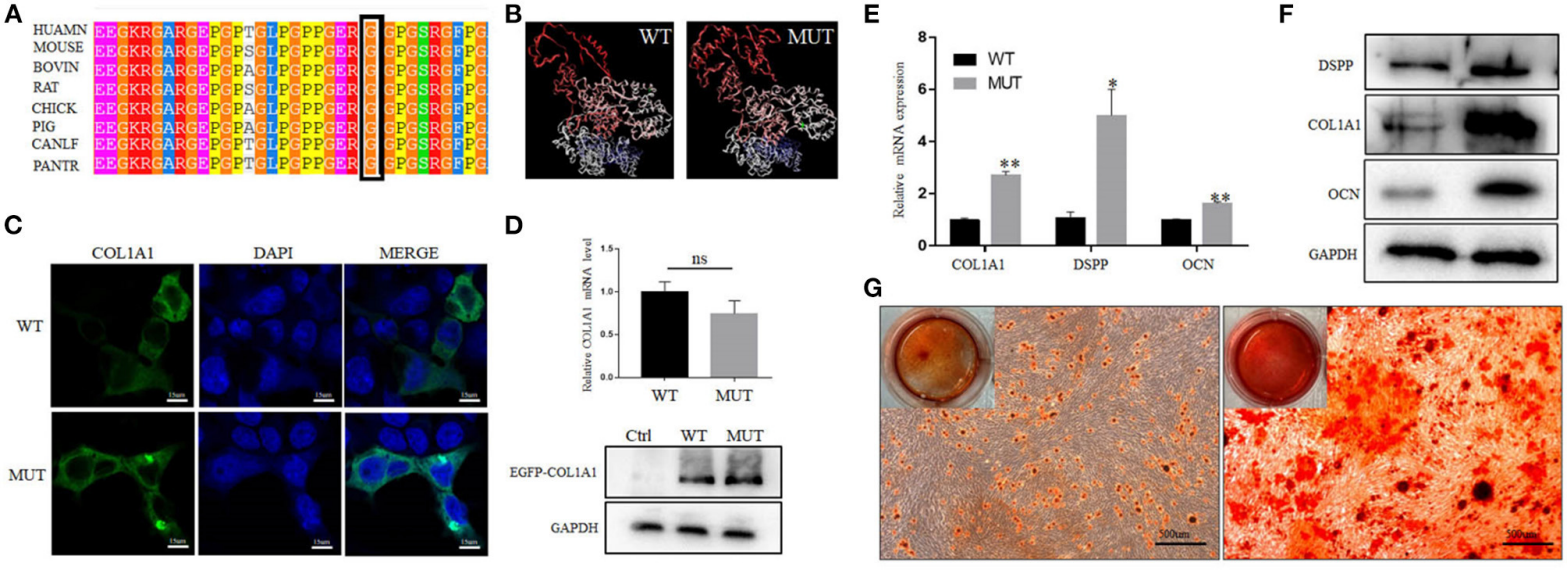
Effect of the mutation on COL1A1 function. **(A)** Conservation analysis of this abnormal variation using Polyphen-2. **(B)** The 3D structure of mutated COL1A1 was different from that of the wild-type (WT) predicted structure using I-TASSER. **(C)** Subcellular localization of COL1A1 in HEK293 cells. The mutant COL1A1 was localized in the cytoplasm similar to the WT protein. **(D)** The mRNA and protein expression levels of COL1A1 in HEK293 cells. Mutant COL1A1 mRNA expression was no different than that of the WT in HEK293 cells, but the mutant COL1A1 protein expression was higher than that of the WT (*P* < 0.05). **(E,F)** Analysis of the mRNA and protein expression of COL1A1 and odontogenic differentiation markers (DSPP and OCN) during the osteogenic differentiation of hDPSCs. Values are represented as means ± SD of three independent experiments (*P < 0.05 and **P < 0.01). **(G)** Representative images from the ARS staining of DPSCs-MUT and DPSCs-CON at the indicated time points after differentiation induction.

Whole-exome sequencing results showed that a novel heterozygous missense variant (c.G1463C, p.G488A) in *COL1A1* exon 22 was found to be the cause of DGI-I in the proband of the family. Sanger sequencing showed that this mutation was not identified in any other members of the family (Figure 3B). Meanwhile, no mutations were detected in the genomic DNA samples from 100 healthy individuals (data not shown). I-TASSER indicated that the *COL1A1* c.1463G>C mutation changed the 3D dimensional structure of the protein, causing changes in the alpha-helix and random coil structures (Figure 4B). The Gly488 position is highly conserved in the other known COL1A1 proteins (Figure 4A), suggesting that it has an important function.

As shown in Figure 4C, there were no differences in the subcellular localization of the MUT compared with the WT protein. In addition, no differences were observed in the levels of mRNA between the cells transfected with the MUT plasmid and those transfected with the WT plasmid. However, western blot analysis revealed that the expression level of mutant COL1A1 protein was increased compared with the WT protein (Figure 4D).

To determine whether the *COL1A1* mutation affected hDPSC differentiation, we detected the changes in the levels of odontogenic-specific mRNA and protein markers, DSPP and OCN, which have been regarded as specific marker of dentinogenesis, in induced hDPSCs using qRT-PCR and western blotting, respectively. The expression levels of *COL1A1, DSPP*, and *OCN* in hDPSCs with the *COL1A1* mutation were significantly higher than those in control hDPSCs (Figure 4E). Moreover, western blotting showed that the expression level of COL1A1, DSPP, and OCN in DGI-I hDPSCs were significantly upregulated compared with those in the control hDPSCs after odontoblastic differentiation (Figure 4F). The results demonstrated that the DGI-I hDPSCs had a higher odontogenic differentiation ability, and the ARS staining results also confirmed this (Figure 4G).

## Discussion

Type I collagen is the most abundant tooth matrix protein and is an ordered heterotrimer that is composed of two α1(I) chains and one α2(I) chain encoded by *COL1A1* and *COL1A2* genes, respectively (Brodsky and Persikov, 2005). Previous studies have shown that the missense substitution of glycine can cause the deformation of the triple helix structure of collagen, instability of the helical structure, and an abnormal synthesis of collagen (Brodsky and Persikov, 2005; Sun et al., 2015; Shi et al., 2019). Notably, the replacement of glycine seems to be linked to the degree of clinical severity of OI (Persikov et al., 2004; Qiu et al., 2018). Similarly, in our studies, the *COL1A1* mutation caused the substitution of glycine by alanine (p.G488A), which can partly explain the fact that the proband only showed a relatively mild phenotype. Our 3D structural analysis also revealed that the influence of the substitution of glycine on the conformation of the protein was relatively local.

From a microstructure point of view, dentine consists of peritubular dentine and intertubular dentine (Ziskind et al., 2011). The tubules of the dentine in the proband were almost completely occluded by peritubular dentine, which reduces the apparent size and number of pores. Previous studies have shown that defective collagen can lead to densely packed mineral particles, causing increased mineralization, which can be a characteristic feature of OI (Fratzl-Zelman et al., 2015). Accordingly, we have also observed that the quality of mineralization in DGI-I dentine was far from satisfactory. First, the dentine hardness and collagen elasticity in the proband were significantly lower than in the control samples (*p* < 0.05), which was consistent with the clinical high brittleness phenomenon (Seyedmahmoud et al., 2017). Meanwhile, the hardness values of the control dentine were in good agreement with the results of previous studies on dentine (Fawzy et al., 2017; Yi et al., 2020). Second, the level of in dentine of patients with DGI-I patient was shown to be lower compared with that in normal dentine, which may have influenced the dentine hardness and mineral content (Park et al., 2020). In addition, we compared the odontogenic abilities of hDPSCs from the proband with a healthy control. The results provided further evidence that the hDPSCs from the mutant proband showed an over-mineralization trend compared with the control, therefore, they may influence the quality of dentine formation.

The dysfunctional condition of odontoblasts, which secrete dentine and predentine, may explain a variety of structural changes in the dentine of patients with DGI-I (Hall et al., 2002; Choi et al., 2010). In our studies, the odontoblasts of the proband had irregular shapes and inverted polarity of odontoblasts, which further confirmed that the *COL1A1* mutation can result in abnormal dentine. In addition, the protein expression of mutant COL1A1 was increased in HEK293, but there is no difference in mRNA levels between the WT plasmid group and the MUT plasmid group. We suspected this might be because the COL1A1 expression was highly regulated at the translational not transcriptional level. The specific molecular mechanisms need to be studied further. In view of the over-mineralization trend of cultured hDPSCs, the abnormal odontoblast morphology, the decrease in the hardness of dentine, and the clinically obliterated dental pulp, we can assume that the *COL1A1* mutation may lead to dysfunctional odontoblasts and disordered matrix deposition and mineralization, eventually leading to complete pulp obliteration (De Coster et al., 2007; Majorana et al., 2010).

In conclusion, we report a novel mutation in exon 22 of *COL1A1*, causing non-syndromic DGI-I in a Chinese family, which expanded the known pathogenic spectrum of the *COL1A1* gene. This study provides new insights into the DGI-I pathogenesis and the possibility of regenerative therapy using hDPSCs from patients.

## Patient's Perspective

After receiving the genetic tests, the proband was grateful to the genetic counseling since her parents and brother were not carriers. Moreover, she did not have any bone problems or other phenotypes associated with OI, only presenting a clinically DGI phenotype. She plans to continue teeth cosmetic repair to achieve more confidence.

## Data Availability Statement

The original contributions presented in the study are included in the article, further inquiries can be directed to the corresponding authors.

## Ethics Statement

The studies involving human participants were reviewed and approved by Ethics Committee of Nanfang Hospital, Southern Medical University, Guangzhou, China. The patients/participants provided their written informed consent to participate in this study. Written informed consent was obtained from the individual(s) for the publication of any potentially identifiable images or data included in this article.

## Author Contributions

YZ, YP, FX, and WY designed the study, performed the experiments, analyzed the data, and wrote the manuscript. ZL, LJ, and JM participated in the experiments and revised the manuscript. All authors have read and approved the final version of the manuscript.

## Conflict of Interest

The authors declare that the research was conducted in the absence of any commercial or financial relationships that could be construed as a potential conflict of interest. The reviewer CD declared a past co-authorship with one of the authors FX to the handling editor.

## Supplementary Material

Supplementary Figure 1 **(A)** Load-displacement curves of the tooth dentine. **(B)** Elemental composition of the normal and affected teeth. The levels of sodium (Na), magnesium (Mg), phosphorus (P) and calcium (Ca) are shown in separate graphs. **(C)** Hardness and modulus of the enamel and dentine of the normal and affected teeth (^*^*P* < 0.05 and ^**^*P* < 0.01).

## Abbreviations

DGI: dentinogenesis imperfecta
DPSC: dental pulp stem cells
OI: osteogenesis imperfecta
SD: standard deviation
SEM: scanning electron microscopy
WT: wild type
MUT: mutant type.
