# Supplementary figures and images for: Case Report: A Novel COL1A1 Missense Mutation Associated With Dentineogenesis Imperfecta Type I
Source: Front Genet. 2021 Jun 23;12:699278. doi: 10.3389/fgene.2021.699278 (PMC8260930; doi:10.3389/fgene.2021.699278)

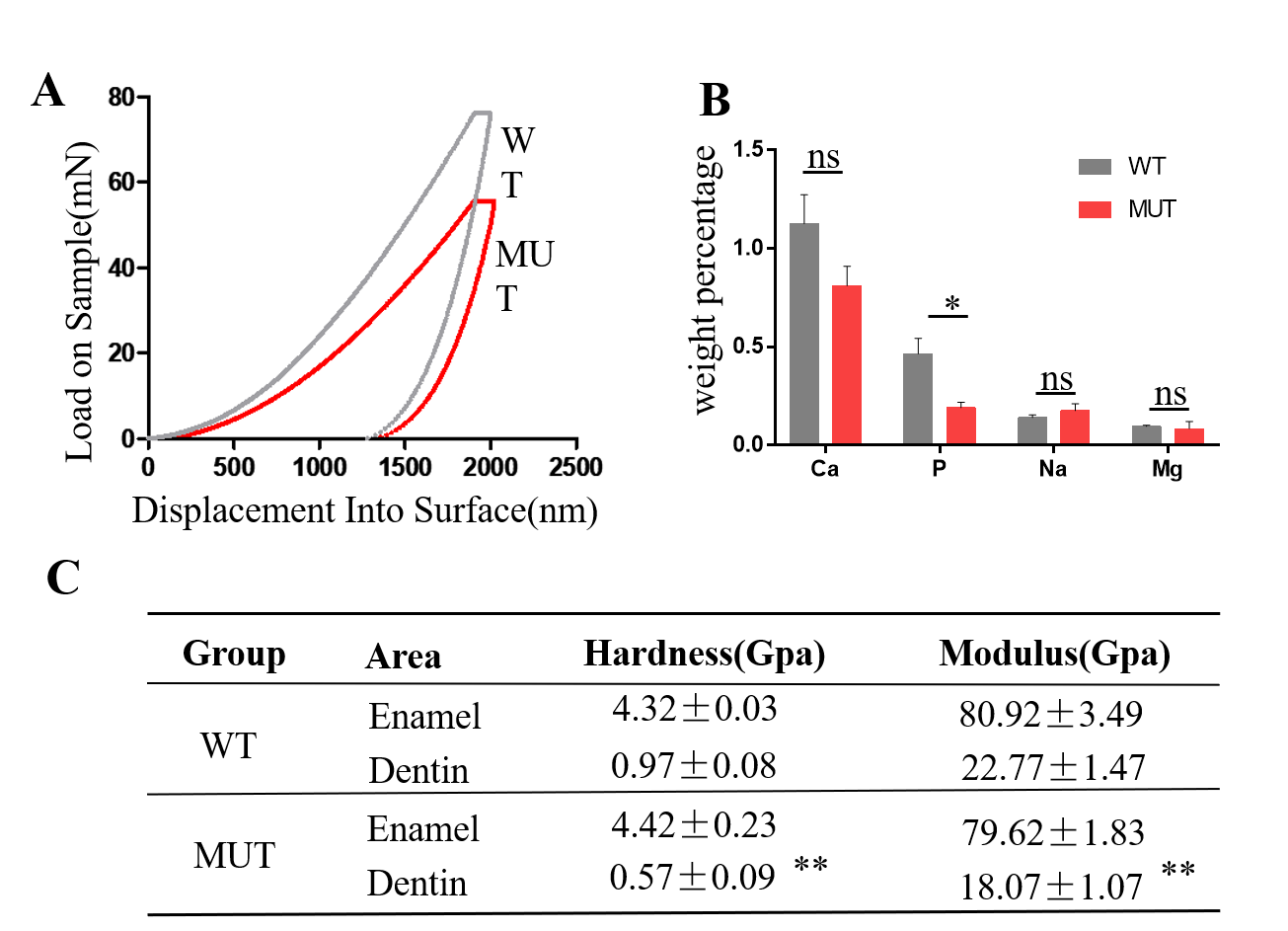

Supplement: Supplementary Figure 1 — (A) Load-displacement curves of the tooth dentine. (B) Elemental composition of the normal and affected teeth. The levels of sodium (Na), magnesium (Mg), phosphorus (P) and calcium (Ca) are shown in separate graphs. (C) Hardness and modulus of the enamel and dentine of the normal and affected teeth (*P < 0.05 and **P < 0.01). [file Image_1.TIF]
